# Characterizing the clinical and sociodemographic profiles of hospitalized adolescents with autism spectrum disorder

**DOI:** 10.1017/gmh.2024.63

**Published:** 2024-05-13

**Authors:** Matan Avrahami, David Haim Ben-Dor, Roy Ratzon, Abraham Weizman, Polina Perlman Danieli

**Affiliations:** 1 Child and Adolescent Division, Geha Mental Health Center, Petah Tikva, Israel; 2 Faculty of Medicine, Tel Aviv University, Tel Aviv, Israel; 3 Laboratory of Molecular and Biological Psychiatry, Felsenstein Medical Research Center, Petah Tikva, Israel; 4 Research Unit, Geha Mental Health Center, Petah Tikva, Israel; 5 Department of Psychiatry, Temerty Faculty of Medicine, University of Toronto, Toronto, ON, Canada; 6 Centre for Addiction and Mental Health, Toronto, ON, Canada

**Keywords:** autism spectrum disorder, intellectual disability, comorbidities

## Abstract

The prevalence of autism spectrum disorder (ASD) is increasing worldwide. Youngsters with ASD demonstrate higher rates of intellectual disabilities (IDs), comorbid psychopathology and psychiatric hospitalizations, compared to children in the general population. This study characterizes the demographics and clinical parameters of adolescent psychiatric inpatients with ASD compared to inpatients without ASD, all hospitalized during the study period. Additionally, within the ASD group, those with ID were compared to those without. The rate of males among participants with ASD was significantly higher than among those without ASD, and the duration of hospitalization was longer. In contrast, the rate of cigarette smoking, major depressive disorder and suicidal thoughts among those with ASD was lower. One-third of those with ASD had moderate to severe ID, about 10% had comorbid epilepsy, and about half of them demonstrated aggressive behavior. Most ASD patients showed significant improvement upon discharge, although the extent of improvement was more prominent among ASD patients with no ID. Our findings, consistent with previous research, indicate that hospitalization is beneficial to youths with ASD, both those with and those without ID. Further studies that include long-term follow-up are needed.

## Impact statement

This study investigates the complex interplay between psychiatric management and clinical outcomes in adolescents with autism spectrum disorder (ASD). By scrutinizing the demographics and clinical profiles of adolescents admitted to psychiatric units, the research underscores the pressing need for tailored interventions and specialized care for this vulnerable population. The findings of this study have significant implications for clinical practice, providing healthcare professionals with insights into managing ASD in psychiatric settings. The study highlights the pervasive nature of comorbidities, particularly intellectual disability (ID), and its impact on clinical presentations and treatment approaches. It also identifies risk factors for hospitalization, such as aggressive behaviors and mood disorders, which can guide early intervention strategies and preventive measures. Furthermore, the study underscores the efficacy of psychiatric hospitalization in effecting meaningful clinical improvements among adolescents with ASD, advocating for the provision of specialized care tailored to their unique needs. The observed reduction in aggressive behaviors and the overall enhancement in quality-of-life post-hospitalization, underscore the pivotal role of specialized psychiatric units in addressing the complex needs of individuals with ASD. The study’s comparison between ASD and non-ASD cohorts unveils disparities in clinical manifestations, challenging prevailing assumptions. Overall, this study sheds light on the common presentation of youths with ASD in inpatient psychiatric settings and supports advocating for holistic, person-centered care that acknowledges the heterogeneity of clinical presentations and addresses the unique needs of individuals with ASD. Its implications are relevant to clinical practice, research and policymaking.

## Introduction

Autism spectrum disorder (ASD) is a heterogeneous neurodevelopmental disorder characterized by impaired social communication and interaction, as well as restricted and repetitive behavior patterns and unusually restricted interests (Lai et al., 2014; Tian et al., 2022). Psychiatric management of children and adolescents with autism spectrum disorder, with or without intellectual disability (ID), is challenging. Children with ASD are 6.61 times more likely to be admitted to a psychiatric hospital than typically developing children (Croen et al., 2006). Parents of 11% of youths with ASD less than 21 years of age reported that their children had been psychiatrically hospitalized at least once (Siegel and Gabriels, 2014). The one-year rate of psychiatric admissions among children with ASD is 1.3% (Croen et al., 2006; Siegel and Gabriels, 2014). Psychiatric units intended especially for patients with ASD or other neurodevelopmental disorders are relatively rare. Moreover, despite the relatively high admission rates among this population, there is little data on the demographics and clinical characteristics of the admitted patients. Children with ASD have higher rates of comorbid psychopathology than their peers without ASD (Al-Beltagi, 2021). Yet, diagnosis is more challenging, and interventions are less effective in children with ASD due to communication difficulties and sometimes due to the presence of ID (McGuire et al., 2015; Al-Beltagi, 2021). Studies showed that 25–70% of individuals with ASD have a comorbid ID of variable severity, while others have some comorbid disability other than cognitive dysfunctions, especially concerning language and behavior (Mefford et al., 2012; Kantzer et al., 2013; Thurm et al., 2019). ASD and ID seem to share genetic substrates and are likely biochemically and molecularly associated (Srivastava and Schwartz, 2014). A recent meta-analysis study evaluated the prevalence of mental health concerns among autistic patients and found high rates of attention deficit and hyperactivity disorder (ADHD) (28%), anxiety disorders (20%), sleep–wake disorders (13%), depressive disorders (11%), obsessive-compulsive disorders (OCD) (9%) and bipolar disorders (5%) (Lai et al., 2019). Moreover, adolescence is a critical period in the emergence of various psychiatric disorders (Périsse et al., 2010). Studies showed that during this period, some individuals with ASD also experienced clinical deterioration that may include cognitive and behavioral regression, catatonic symptoms, psychosis and epileptic seizures (Smile, 2016; Liu et al., 2022). One study reported that 10.9% of ASD patients showed a progressive deterioration during adolescence, which began with “a loss of language skills associated with inertia and decreasing activity followed by a general intellectual decline.” In three of these patients, epileptic fits accompanied deterioration, and one patient had severe OCD (Ghaziuddin, 2021). Billstedt and colleagues estimated the rate of progressive deterioration in a group of 120 patients with ASD to be as high as 20% during adolescence (Billstedt et al., 2005). They noted that in 12 (10%) out of these, the “deterioration appeared to be permanent.”

Studies found that the highest risk factors for hospitalization due to ASD are aggressive behaviors, self-endangerment, comorbidity of mood disorders, sleep problems, comorbid OCD and older age at diagnosis (Siegel and Gabriels, 2014; Righi et al., 2018). The risk of admission increases with age of the individual with ASD (Ozbaran et al., 2022). A study of patients with ASD and ID hospitalized due to behavioral or cognitive regression found environmental triggers (lack of proper care, adjustment disorder) in half the cases, physical problems such as seizures in one-third, and diagnoses of various psychiatric disorders, such as depressive and bipolar disorder, in the rest (Périsse et al., 2010). The presence of psychopathology in the mother and siblings of patients with ASD was found to be significantly higher in this group (61.8% and 26.5%, respectively), while only 23.5% of fathers were found to have psychopathology. The most common psychopathologies were major depressive disorder in mothers, ADHD in fathers and ASD in siblings (Ozbaran et al., 2022). Some factors were found to have a positive effect on ASD patients, such as living in an area with easy access to specialized health care providers and being female. Regular periods of relief and rest (respite) for the caretakers were found to be a protective factor as well (Mandell et al., 2012).

Prevalence of ASD diagnosis in the US has increased from 3 in 10,000 children in the 1980s to 1 in 44 children in 2018 (Maenner et al., 2021). This seems to be true for other Western countries as well (Li et al., 2022). Explanations for this rise include improved clinical awareness of the disorder leading to earlier and more accurate diagnoses and true increase in incidence (Thurm et al., 2019; Maenner et al., 2021). A similar increase is seen in Israel between the years 2000–2011, from 1 in 2,000 to 1 in 200 (Davidovitch et al., 2013; Raz et al., 2015).

The aims of the current study were as follows: 1) to characterize the clinical factors and demographics of adolescents with ASD admitted to a psychiatric inpatient department; 2) to compare sociodemographic and clinical characteristics of patients with ASD and comorbid ID (ASD + ID) to those of patients with ASD and no ID (ASD-ID); 3) to compare sociodemographic and clinical characteristics of patients with ASD to those of patients without ASD (non-ASD), hospitalized in the department during the same time period.

## Methods

Data for this study were retrieved from the electronic medical records (EMRs) and medical hard copy files of a major mental health center in central Israel. It included all admissions to the Child and Adolescent Division (age 12–20 years old) between January 1, 2010 and December 31, 2015. The ASD group (*n* = 64) included patients diagnosed with childhood autism according to the DSM-5 criteria. To establish an ASD diagnosis in Israel, a child or adolescent is assessed by two specialists, both following the DSM-5 criteria: 1) A board-certified child and adolescent psychiatrist, and 2) a developmental, educational or clinical psychologist who, additionally, uses the Autism Diagnostic Observation Schedule (ADOS; Lord et al., 1989), the Autism Diagnostic Interview, Revised (ADI-R; Lord et al., 1994), and a cognitive assessment. The scores of the latter tools are stored at the Israeli National Insurance Agency.

The ASD group was further divided into ASD + ID (*n* = 18) and ASD-ID (*n* = 46). Various sociodemographic and clinical characteristics were compared between the two ASD subgroups. These characteristics are described in detail later in the Methods section.

Various sociodemographic and clinical characteristics from the whole ASD group (*n* = 64) were compared to a group (*n* = 648) of hospitalized children who have no ASD (non-ASD).

The institutional review board approved the study and waived the need for written informed consent by the participants due to the study’s retrospective nature and de-identified data.

Demographics (age, sex, family structure), age at ASD diagnosis, DSM-5 diagnosis at discharge, place of living and type of school prior to admission and at discharge were collected from the EMRs and the hard copy files. Also collected were data on the presence of affective or psychotic symptoms, presence of ID and epilepsy, family history of affective or psychotic disorders, suicidal thoughts, suicide attempts, or non-suicidal self-injury (NSSI) at admission, behaviors that pose a danger to self or others (i.e., aggressiveness), as well as length of hospital stay, number of previous admissions and antipsychotics the patient was receiving at admission. Data on place of living and school placement following discharge were also collected. Scores of the Clinical Global Impression – Severity (CGI-S) scale at admission and the Clinical Global Impression – Improvement (CGI-I) scale at discharge were determined retrospectively, based on the data in the patient files, by three child psychiatrists (MA, RR, PP) with the final scores determined by consensus. All patients hospitalized in the department receive ongoing psychiatric care along with various individually tailored psychosocial interventions such as special school programs according to the patient’s needs, animal therapy, group therapy, individual therapy and parental guidance. These are provided regardless of length of stay.

Statistical analysis was performed using IBM’s SPSS for Windows ver. 24.0 (IBM Corp., Armonk, New York, USA). The ASD group was further analyzed according to the presence or absence of ID comorbidity. T-test and Chi-square test were used as appropriate. Statistical significance was set at *p* < 0.05. Significant results underwent Bonferroni correction.

## Results

### Patients with ASD

Sociodemographics and clinical characteristics of ASD patients are shown in Tables 1–4.

**Table 1.** Sociodemographics and clinical responsiveness to treatment of ASD patients with ID and without ID

	Total ASD (*N* = 64)	ASD + ID (*N* = 18)	ASD-ID (*N* = 46)	*t*-test	*p*-value
	Mean (SD)	Mean (SD)	Mean (SD)		
Age (years)	15.1 (1.8)	14.7 (2.1)	15.3 (1.6)	1.2	0.2
Length of hospitalization (days)	89.4 (104)	71.9 (92.3)	96.2 (108.3)	0.84	0.4
Number of previous admissions	0.58 (0.94)	0.83 (1.15)	0.48 (0.83)	−1.37	0.2
Age at ASD diagnosis		6.85 (4.35)	9.24 (5.18)	1.68	0.1
CGI–severity at admission		5.44 (0.78)	4.87 (1)	−2.07	0.04^*^
CGI–improvement at discharge		2.44 (1.1)	1.85 (0.92)	−2.2	0.03^*^

ASD = autism spectrum disorder; ID = intellectual disability; ASD + ID = ASD patients with ID; ASD-ID = ASD without ID; CGI = clinical global impression. *Non-significant following Bonferroni correction for multiple comparisons.

#### Clinical improvement during hospitalization

**Figure 1.**
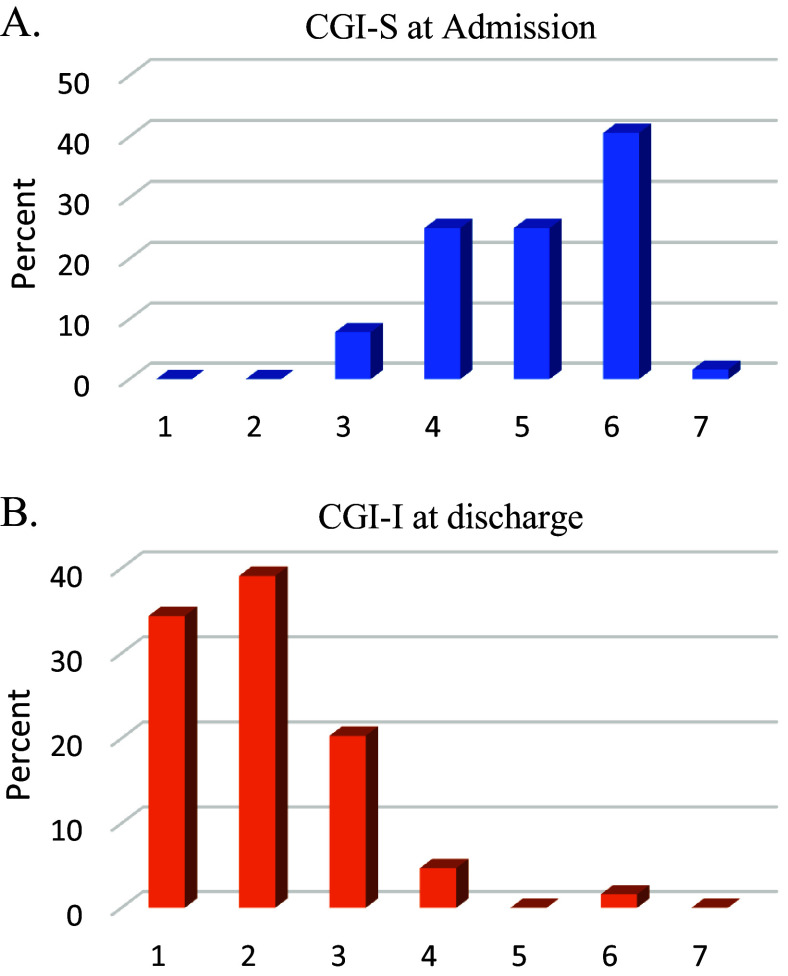
The distribution (%) of CGI-S and CGI-I scores in the ASD group. CGI-S = Clinical Global Impression – Severity, CGI-I = Clinical Global Impression – Improvement. A) CGI-S: 1 = normal, not at all ill; 2 = borderline mentally ill; 3 = mildly ill; 4 = moderately ill; 5 = markedly ill; 6 = severely ill; 7 = among the most extremely ill patients. B) CGI-I: 1 = very much improved; 2 = much improved; 3 = minimally improved; 4 = no change; 5 = minimally worse; 6 = much worse; 7 = very much worse.

Figure 1A and B presents the retrospective assessment of the distribution of comorbid psychopathology degrees in patients with ASD, as measured by the CGI-S and CGI-I scores. It appears that at admission, most of the patients with ASD (80%) had a CGI-S score in the range of 4–6. Following hospitalization, significant improvement was achieved in a substantial proportion (85%) of the population with ASD, namely, CGI-I at discharge was in the range of 1–3.

#### Post-hospitalization care and education

At discharge, eight patients (12.5%) with ASD were placed in a facility for youths with ASD. Three youngsters (4.7%) who had not attended special education programs in the community prior to hospitalization were now placed in such programs. The majority (*n* = 53; 82.8%) returned to the special education programs in the community they had attended prior to hospitalization.

### Comparison between individuals with ASD and comorbid ID (ASD + ID) vs. individuals with ASD and no ID (ASD-ID)

Tables 1 and 2 present sociodemographics and clinical characteristics of the ASD + ID and ASD-ID groups. There were 18 ASD + ID patients and 46 ASD-ID patients. The comparison between the groups shows that ASD + ID patients had significantly higher CGI-S scores at admission and worse CGI-I scores at discharge than ASD-ID patients. However, after Bonferroni correction for multiple comparisons, these differences did not reach statistical significance. Other parameters like the presence of epilepsy, maintenance of antipsychotics at admission, need for restraint or seclusion and self-injurious and aggressive behaviors were not different between the two groups.

**Table 2.** Clinical characteristics of ASD patients with ID and without ID

	Total ASD (*N* = 64)	ASD + ID (*N* = 18)	ASD-ID (*N* = 46)	*χ*^2^/FET	*p*-value
	n	*n* (%)	*n* (%)		
Maintained on antipsychotics at admission	40	11 (61.1)	29 (63)	0.02	0.9
Restraint, seclusion	9	3 (16.7)	6 (13)	0.37	0.7
Self–injurious behavior during hospitalization	12	5 (27.8)	7 (15.2)	0.29	0.25
Aggressive behavior during hospitalization	35	11 (61.1)	24 (52.2)	0.42	0.52
Epilepsy	5	3 (16.7)	2 (4.3)	2.7	0.1

ASD = autism spectrum disorder; ID = intellectual disability; ASD + ID = ASD patients with ID; ASD-ID = ASD without ID; FET = Fisher’s exact test.

### Comparison of individuals with ASD vs. individuals with no ASD (non-ASD)

The study included 64 inpatients with ASD and 648 inpatients without ASD. Their demographics and clinical parameters are shown in Tables 3 and 4. ASD and non-ASD inpatients were of similar age (averaging 15 years old). The ASD group demonstrated longer hospitalization periods than the non-ASD group. The rate of males in the ASD group was higher, and the rate of cigarette smoking was lower compared to non-ASDs. The rates of major depressive episodes and suicidal thoughts at admission were found to be lower in the ASD group, while rates of previous admissions (*p* = 0.001) were higher in the ASD group.

**Table 3.** Comparison of age and clinical characteristics between participants with ASD and those without

	ASD (n = 64)	Non-ASD (n = 648)	Student’s *t*-test	*p*-value
	Mean (SD)	Mean (SD)		
Age (years)	15.1 (1.8)	15.2 (1.8)	0.41	0.682
Length of hospitalization (days)	89.4 (104)	54.5 (74)	−3.5	**0.001** ^*^
Number of previous admissions	0.58 (0.94)	0.3 (0.81)	−2.6	0.01

ASD = autism spectrum disorder. *Remains significant following Bonferroni correction.

**Table 4.** Comparison of sex rates and rates of clinical characteristics between participants with ASD and those without

	ASD (*n* = 64)	Non-ASD (*n* = 648)	*χ*^2^	*p*-value
	*N* (%)	*N* (%)		
Male sex	51 (79.7)	285 (44)	29.8	<0.001^*^
Cigarette Smokers	6 (9.4)	164 (25.3)	14.3	0.001^*^
Family history of affective disorders	5 (10.6)	83 (16.7)	1.2	0.28
Family history of psychotic disorders	6 (12.8)	50 (10)	0.36	0.55
Compulsory admissions	9 (14.1)	158 (24.5)	3.52	0.06
Restraint, seclusion	9 (14.1)	44 (6.8)	4.47	0.03
Major depressive episodes	4 (6.3)	248 (38.6)	25.9	<0.001^*^
Manic episodes	8 (12.5)	62 (9.6)	0.55	0.46
Psychotic episodes	11 (17.2)	142 (22)	0.81	0.37
Mixed episodes	1 (1.6)	7 (1.1)	0.12	0.73
Suicidal thoughts at admission	11 (19.3)	291 (46.6)	15.8	<0.001*
Suicidal attempts prior to admission	3 (4.7)	111 (17.2)	6.74	0.009
NSSI prior to admission	6 (9.4)	135 (21.1)	5	0.026

ASD = autism spectrum disorder; NSSI = non-suicidal self-injury. *Remains significant following Bonferroni correction.

## Discussion

### Comparison of individuals with ASD and comorbid ID versus individuals with ASD and no ID

The ASD group was divided into the ASD + ID group and the ASD-ID group. No significant differences in sociodemographics and clinical parameters were observed between the two groups. Adults diagnosed with ASD + ID have been reported to demonstrate aggression more frequently than adults with ID alone (Tsakanikos et al., 2007). Farmer et al. (2015) reported high rates of aggression in children with ASD towards both caregivers and non-caregivers. In our study on adolescents, aggressive behavior was observed in more than half of the inpatients with ASD. Aggression clearly has a negative impact on the quality of life in children with ASD, leading to impaired social relationships, placement in special education programs and institutes, including residential schools, use of physical intervention and increased risk of being victimized (Fitzpatrick et al., 2016).

The majority (62%) of the ASD group in our study had been maintained on antipsychotics prior to admission, most likely due to irritability, aggressive behaviors, or psychotic symptoms. Antipsychotics are typically used to treat associated comorbidities, such as schizophrenia and behavior disorders (Lee et al., 2018). A systematic review by Pillay and colleagues suggested that antipsychotics may be helpful in improving symptoms of irritability and possibly stereotyped behaviors. Evidence was sparse for several patient- and family-important outcomes, such as health-related quality of life, involvement with the legal system and school performance (Pillay et al., 2017). Antipsychotics were proven to reduce hyperactivity, attention deficit, opposition and disruptive behaviors, and slightly decrease restricted and repetitive interests and behaviors, obsession and compulsion in children and adolescents with ASD. Moreover, antipsychotics seem to slightly reduce emotional dysregulation/irritability and positively influence global functioning (D’Alò et al., 2021).

A high rate of comorbid ID is present in inpatients with ASD, as was the case in our sample (Melvin et al., 2022). As indicated above, more than half of our sample with ASD demonstrated aggressive behavior. One-third of the patients demonstrated moderate to severe ID, and about 10% had comorbid epilepsy. The ASD + ID tended to have mild-to-borderline ID and were of similar age as the ASD-ID. In fact, genetic variants associated with ASD are often also associated with ID, validating both the phenotypic and genotypic overlap between these conditions (Zhu et al., 2014; Casanova et al., 2016). In addition, the rate of genetic variants associated with ASD is significantly higher in the presence of comorbid ID (Sanders et al., 2015). ASD genetics are mostly complex, involving multiple genes with small contributions to illness expression. Genotyping and computing genotypic risk scores could potentially aid future diagnostic assessments of ASD by capturing the cumulative effects of sequence number variants and copy number variants, which elevate the risk for ASD and other related neuropsychiatric and neurodevelopmental conditions (An et al., 2018; LaBianca et al., 2021; Cirnigliaro et al., 2023; Fusar-Poli et al., 2023).

By the time people reach adolescence, the prevalence of epilepsy in the general population is between 1% and 2%, in contrast to it being between 20% and 30% in individuals with ASD (Tuchman and Cuccaro, 2011; Besag, 2018). In ASD, two peak periods of epilepsy onset have been described, one in early childhood and the second in adolescence (Parmeggiani et al., 2010).

#### The effect of hospitalization on the ASD group

Most CGI-S scores obtained at admission ranged from moderately ill to severely ill.

At discharge, assessment with CGI-I showed meaningful clinical improvement (“very much” to “much improvement”) in about 70% of the ASD group. Thus, most patients benefited from the hospitalization, as was also reflected by the majority being able to return to their families and their pre-hospitalization non-residential special education programs (Figure 1A and B). Only three patients had to be transferred from their previous in-the-community programs to residential ASD facilities.

Several studies provided evidence of improvements in mental health, social functioning, behavior and forensic risk after inpatient admission (Melvin et al., 2022). Treatment in a psychiatric inpatient unit specializing in ASD and ID was associated with a significant reduction in aggressive, self-injurious and tantrum-like behaviors in children with ASD. These benefits were sustained at a follow-up 2 months post-discharge (Siegel and Gabriels, 2014).

### Comparison of individuals with ASD versus individuals with no ASD

In our study, about 80% of the patients with ASD were males, which is in line with previous studies. Ozbaran et al. (2022) found that there were more male inpatients with ASD than female ones. Other previous studies showed that male sex dominance is seen in hospitalized ASD patients (Kuriakose et al., 2018; Taylor et al., 2019). ASD male-to-female ratios show striking variability, even in epidemiological studies that implemented equivalent inclusion criteria and recruitment methods. These ratios range between 8-to-1 and 2-to-1 (Loomes et al., 2017). Loomes et al. (2017) claimed that the male-to-female ratio among children meeting criteria for ASD is closer to 3:1, and not 4:1, as is stated in the DSM-5. Thus, it seems that female adolescents with autism are at greater risk than males of having their ASD overlooked, misdiagnosed, or identified late (Russell et al., 2011; Mandy and Tchanturia, 2015).

Surprisingly, in our study, the rates of major depressive episodes and suicidal thoughts at admission were found to be notably lower in the ASD group than in the non-ASD group. In a study conducted by Ozbaran et al. (2022), the rate of suicide attempts was also found to be significantly lower in the ASD group. These findings may be relevant to hospitalized patients with ASD but not to the general population of individuals with ASD and no ID (Newell et al., 2023). In general, depression is one of the most prevalent co-morbid mental health conditions affecting up to 50% of individuals with ASD during their lifetime (Hedley et al., 2017), and thus, patients with high-functioning ASD are more likely to experience suicide attempts (Karakoç Demirkaya et al., 2016; Newell et al., 2023). According to parents’ reports, talk of death or suicide was common in 22% of youth with ASD assessed during an inpatient psychiatric admission (Horowitz et al., 2018). Thus, the lower rates of major depressive episodes and suicidal thoughts in our study may suggest detection bias, as behavioral deterioration leading to psychiatric admission in this population may be the result of underdiagnosed depression and suicidality. Consistent with previous reports, the group of adolescents with ASD was found to be smoking cigarettes significantly less than their non-ASD peers (Bejerot and Nylander, 2003; Mattila et al., 2010).

### Limitations

The major limitations of the current study are the relatively small sample size, its retrospective-naturalistic nature and lack of follow-up. In addition, the study does not include a control group for the CGI-S and CGI-I results and finally, no rating scale that is specific for ASD, is used in the study.

## Conclusion

Our study compared sociodemographics and clinical characteristics of adolescent inpatients with ASD, between those with ID and those without, and compared the patients with ASD to patients without ASD. These analyses are scarce in the literature.

Surprisingly, the rate of aggressive episodes, as measured by restraint and seclusion events, was not significantly different between those with ASD and those without. Also surprising was the finding that patients with ASD demonstrated lower rates of depression and suicidality. Consistent with previous studies, in our study, patients with ASD were found to smoke cigarettes significantly less than those without. Since significant clinical improvement was observed in our sample of adolescents with ASD following hospitalization, it seems that hospitalization is beneficial to those patients. As mentioned in the limitations, this study lacked follow-up, which should be done in future studies in order to substantiate the long-term benefits of hospitalization for ASD youths.

## Data Availability

Due to the need for confidentiality, data cannot be published on a public resource and are available following reasonable request, from the corresponding author.

## References

[r1] Al-Beltagi M (2021) Autism medical comorbidities. World Journal of Clinical Pediatrics 10(3), 15–28. 10.5409/wjcp.v10.i3.15. 33972922 PMC8085719

[r2] An JY, Lin K, Zhu L, Werling DM, Dong S, Brand H, Wang HZ, Zhao X, Schwartz GB, Collins RL, Currall BB, Dastmalchi C, Dea J, Duhn C, Gilson MC, Klei L, Liang L, Markenscoff-Papadimitriou E, Pochareddy S, Ahituv N, Buxbaum JD, Coon H, Daly MJ, Kim YS, Marth GT, Neale BM, Quinlan AR, Rubenstein JL, Sestan N, State MW, Willsey AJ, Talkowski ME, Devlin B, Roeder K and Sanders SJ (2018) Genome-wide de novo risk score implicates promoter variation in autism spectrum disorder. Science 362(6420). 10.1126/science.aat6576. PMC6432922 30545852

[r3] Bejerot S and Nylander L (2003) Low prevalence of smoking in patients with autism spectrum disorders. Psychiatry Research 119(1–2), 177–182. 10.1016/s0165-1781(03)00123-9. 12860373

[r4] Besag FM (2018) Epilepsy in patients with autism: Links, risks and treatment challenges. Neuropsychiatric Disease and Treatment 14, 1–10. 10.2147/ndt.S120509. 29296085 PMC5739118

[r5] Billstedt E, Gillberg C and Gillberg C (2005) Autism after adolescence: Population-based 13-to 22-year follow-up study of 120 individuals with autism diagnosed in childhood. Journal of Autism and Developmental Disorders 35(3), 351–360. 10.1007/s10803-005-3302-5. 16119476

[r6] Casanova EL, Sharp JL, Chakraborty H, Sumi NS and Casanova MF (2016) Genes with high penetrance for syndromic and non-syndromic autism typically function within the nucleus and regulate gene expression. Molecular Autism 7(1), 1–17. 10.1186/s13229-016-0082-z. 26985359 PMC4793536

[r7] Cirnigliaro M, Chang TS, Arteaga SA, Pérez-Cano L, Ruzzo EK, Gordon A, Bicks LK, Jung JY, Lowe JK, Wall DP and Geschwind DH (2023) The contributions of rare inherited and polygenic risk to ASD in multiplex families. Proceedings of the National Academy of Sciences of the United States of America 120(31), e2215632120. 10.1073/pnas.2215632120. 37506195 PMC10400943

[r8] Croen LA, Najjar DV, Ray GT, Lotspeich L and Bernal P (2006) A comparison of health care utilization and costs of children with and without autism spectrum disorders in a large group-model health plan. Pediatrics 118(4), e1203–1211. 10.1542/peds.2006-0127. 17015508

[r9] D’Alò GL, De Crescenzo F, Amato L, Cruciani F, Davoli M, Fulceri F, Minozzi S, Mitrova Z, Morgano GP, Nardocci F, Saulle R, Schünemann HJ, Scattoni ML, Tancredi R, Massagli A, Valeri G, Cappa C, Buono S, Arduino GM, Zuddas A, Reali L, Molteni M, Felici C, Cordò C, Venturini L, Bellosio C, Di Tommaso E, Biasci S, Duff CM, Vecchi S and On Behalf of the IWG (2021) Impact of antipsychotics in children and adolescents with autism spectrum disorder: A systematic review and meta-analysis. Health and Quality of Life Outcomes 19(1), 33. 10.1186/s12955-021-01669-0. 33494757 PMC7831175

[r10] Davidovitch M, Hemo B, Manning-Courtney P and Fombonne E (2013) Prevalence and incidence of autism spectrum disorder in an Israeli population. Journal of Autism and Developmental Disorders 43(4), 785–793. 10.1007/s10803-012-1611-z. 22836322

[r11] Farmer C, Butter E, Mazurek MO, Cowan C, Lainhart J, Cook EH, DeWitt MB and Aman M (2015) Aggression in children with autism spectrum disorders and a clinic-referred comparison group. Autism 19(3), 281–291. 10.1177/1362361313518995. 24497627 PMC4331245

[r12] Fitzpatrick SE, Srivorakiat L, Wink LK, Pedapati EV and Erickson CA (2016) Aggression in autism spectrum disorder: Presentation and treatment options. Neuropsychiatric Disease and Treatment 12, 1525–1538. 10.2147/ndt.S84585. 27382295 PMC4922773

[r13] Fusar-Poli L, Rodolico A, Martinez M, Fichera C, Lin BD, Basadonne I, Concerto C, Aguglia E, Guloksuz S and Signorelli MS (2023) The association between polygenic risk scores for mental disorders and social cognition: A scoping review. Journal of Psychiatric Research 164, 389–401. 10.1016/j.jpsychires.2023.06.029. 37418886

[r14] Ghaziuddin M (2021) Catatonia: A common cause of late regression in autism. Frontiers in Psychiatry 12, 674009. 10.3389/fpsyt.2021.674009. 34777033 PMC8585308

[r15] Hedley D, Uljarević M, Wilmot M, Richdale A and Dissanayake C (2017) Brief report: Social support, depression and suicidal ideation in adults with autism spectrum disorder. Journal of Autism and Developmental Disorders 47(11), 3669–3677. 10.1007/s10803-017-3274-2. 28861661

[r16] Horowitz LM, Thurm A, Farmer C, Mazefsky C, Lanzillo E, Bridge JA, Greenbaum R, Pao M, Siegel M, Siegel M, Erickson C, Gabriels RL, Kaplan D, Mazefsky C, Morrow EM, Righi G, Santangelo SL, Wink L, Benevides J, Beresford C, Best C, Bowen K, Dechant B, Flis T, Gastgeb H, Geer A, Hagopian L, Handen B, Klever A, Lubetsky M, MacKenzie K, Meservy Z, McGonigle J, McGuire K, McNeil F, Montrenes J, Palka T, Pedapati E, Pedersen KA, Peura C, Pierri J, Rogers C, Rossman B, Ruberg J, Sannar E, Small C, Stuckey N, Troen B, Tylenda B, Verdi M, Vezzoli J, Williams D, Williams D, for the A and Developmental Disorders Inpatient Research C (2018) Talking about death or suicide: Prevalence and clinical correlates in youth with autism spectrum disorder in the psychiatric inpatient setting. Journal of Autism and Developmental Disorders 48(11), 3702–3710. 10.1007/s10803-017-3180-7. 28624965 PMC7410502

[r17] Kantzer AK, Fernell E, Gillberg C and Miniscalco C (2013) Autism in community pre-schoolers: Developmental profiles. Research in Developmental Disabilities 34(9), 2900–2908. 10.1016/j.ridd.2013.06.016. 23816626

[r18] Karakoç Demirkaya S, Tutkunkardaş MD and Mukaddes NM (2016) Assessment of suicidality in children and adolescents with diagnosis of high functioning autism spectrum disorder in a Turkish clinical sample. Neuropsychiatric Disease and Treatment 12, 2921–2926. 10.2147/ndt.S118304. 27956832 PMC5113915

[r19] Kuriakose S, Filton B, Marr M, Okparaeke E, Cervantes P, Siegel M, Horwitz S and Havens J (2018) Does an autism Spectrum disorder care pathway improve care for children and adolescents with ASD in inpatient psychiatric units? Journal of Autism and Developmental Disorders 48(12), 4082–4089. 10.1007/s10803-018-3666-y. 29971653

[r20] LaBianca S, LaBianca J, Pagsberg AK, Jakobsen KD, Appadurai V, Buil A and Werge T (2021) Copy number variants and polygenic risk scores predict need of care in autism and/or ADHD families. Journal of Autism and Developmental Disorders 51(1), 276–285. 10.1007/s10803-020-04552-x. 32462456

[r21] Lai MC, Kassee C, Besney R, Bonato S, Hull L, Mandy W, Szatmari P and Ameis SH (2019) Prevalence of co-occurring mental health diagnoses in the autism population: A systematic review and meta-analysis. Lancet Psychiatry 6(10), 819–829. 10.1016/s2215-0366(19)30289-5. 31447415

[r22] Lai MC, Lombardo MV and Baron-Cohen S (2014) Autism. Lancet 383(9920), 896–910. 10.1016/s0140-6736(13)61539-1. 24074734

[r23] Lee ES, Vidal C and Findling RL (2018) A focused review on the treatment of pediatric patients with atypical antipsychotics. Journal of Child and Adolescent Psychopharmacology 28(9), 582–605. 10.1089/cap.2018.0037. 30312108

[r24] Li YA, Chen ZJ, Li XD, Gu MH, Xia N, Gong C, Zhou ZW, Yasin G, Xie HY, Wei XP, Liu YL, Han XH, Lu M, Xu J and Huang XL (2022) Epidemiology of autism spectrum disorders: Global burden of disease 2019 and bibliometric analysis of risk factors. Frontiers in Pediatrics 10, 972809. 10.3389/fped.2022.972809. 36545666 PMC9760802

[r25] Liu X, Sun X, Sun C, Zou M, Chen Y, Huang J, Wu L and Chen WX (2022) Prevalence of epilepsy in autism spectrum disorders: A systematic review and meta-analysis. Autism 26(1), 33–50. 10.1177/13623613211045029. 34510916

[r26] Loomes R, Hull L and Mandy WPL (2017) What is the male-to-female ratio in autism spectrum disorder? A systematic review and meta-analysis. Journal of the American Academy of Child and Adolescent Psychiatry 56(6), 466–474. 10.1016/j.jaac.2017.03.013. 28545751

[r27] Lord C, Rutter M, Goode S, Heemsbergen J, Jordan H, Mawhood L and Schopler E (1989) Autism diagnostic observation schedule: A standardized observation of communicative and social behavior. Journal of Autism and Developmental Disorders 19(2), 185–212. 10.1007/bf02211841. 2745388

[r28] Lord C, Rutter M and Le Couteur A (1994) Autism diagnostic interview-revised: A revised version of a diagnostic interview for caregivers of individuals with possible pervasive developmental disorders. Journal of Autism and Developmental Disorders 24(5), 659–685. 10.1007/bf02172145. 7814313

[r29] Maenner MJ, Shaw KA, Bakian AV, Bilder DA, Durkin MS, Esler A, Furnier SM, Hallas L, Hall-Lande J, Hudson A, Hughes MM, Patrick M, Pierce K, Poynter JN, Salinas A, Shenouda J, Vehorn A, Warren Z, Constantino JN, DiRienzo M, Fitzgerald RT, Grzybowski A, Spivey MH, Pettygrove S, Zahorodny W, Ali A, Andrews JG, Baroud T, Gutierrez J, Hewitt A, Lee LC, Lopez M, Mancilla KC, McArthur D, Schwenk YD, Washington A, Williams S and Cogswell ME (2021) Prevalence and characteristics of autism spectrum disorder among children aged 8 years – autism and developmental disabilities monitoring network, 11 sites, United States, 2018. MMWR Surveillance Summaries 70(11), 1–16. 10.15585/mmwr.ss7011a1. PMC8639024 34855725

[r30] Mandell DS, Xie M, Morales KH, Lawer L, McCarthy M and Marcus SC (2012) The interplay of outpatient services and psychiatric hospitalization among medicaid-enrolled children with autism spectrum disorders. Archives of Pediatrics & Adolescent Medicine 166(1), 68–73. 10.1001/archpediatrics.2011.714. 22213753 PMC4764986

[r31] Mandy W and Tchanturia K (2015) Do women with eating disorders who have social and flexibility difficulties really have autism? A case series. Molecular Autism 6(1), 6. 10.1186/2040-2392-6-6. 26056560 PMC4459459

[r32] Mattila ML, Hurtig T, Haapsamo H, Jussila K, Kuusikko-Gauffin S, Kielinen M, Linna SL, Ebeling H, Bloigu R, Joskitt L, Pauls DL and Moilanen I (2010) Comorbid psychiatric disorders associated with Asperger syndrome/high-functioning autism: A community- and clinic-based study. Journal of Autism and Developmental Disorders 40(9), 1080–1093. 10.1007/s10803-010-0958-2. 20177765

[r33] McGuire K, Erickson C, Gabriels RL, Kaplan D, Mazefsky C, McGonigle J, Meservy J, Pedapati E, Pierri J, Wink L and Siegel M (2015) Psychiatric hospitalization of children with autism or intellectual disability: Consensus statements on best practices. Journal of the American Academy of Child and Adolescent Psychiatry 54(12), 969–971. 10.1016/j.jaac.2015.08.017. 26598469 PMC4847534

[r34] Mefford HC, Batshaw ML and Hoffman EP (2012) Genomics, intellectual disability, and autism. The New England Journal of Medicine 366(8), 733–743. 10.1056/NEJMra1114194. 22356326 PMC4107681

[r35] Melvin CL, Barnoux M, Alexander R, Roy A, Devapriam J, Blair R, Tromans S, Shepstone L and Langdon PE (2022) A systematic review of in-patient psychiatric care for people with intellectual disabilities and/or autism: Effectiveness, patient safety and experience. BJPsych Open 8(6), e187. 10.1192/bjo.2022.571. 36268640 PMC9634562

[r36] Newell V, Phillips L, Jones C, Townsend E, Richards C and Cassidy S (2023) A systematic review and meta-analysis of suicidality in autistic and possibly autistic people without co-occurring intellectual disability. Molecular Autism 14(1), 12. 10.1186/s13229-023-00544-7. 36922899 PMC10018918

[r37] Ozbaran B, Kose S, Barankoglu I and Dogan N (2022) Inpatient care unit in children and adolescents with autism spectrum disorder: Benefits, difficulties, and conditions of hospitalization. The Journal of Nervous and Mental Disease 210(3), 206–211. 10.1097/nmd.0000000000001429. 34643184

[r38] Parmeggiani A, Barcia G, Posar A, Raimondi E, Santucci M and Scaduto MC (2010) Epilepsy and EEG paroxysmal abnormalities in autism spectrum disorders. Brain and Development 32(9), 783–789. 10.1016/j.braindev.2010.07.003. 20691552

[r39] Périsse D, Amiet C, Consoli A, Thorel MV, Gourfinkel-An I, Bodeau N, Guinchat V, Barthélémy C and Cohen D (2010) Risk factors of acute behavioral regression in psychiatrically hospitalized adolescents with autism. Journal of Canadian Academy of Child and Adolescent Psychiatry 19(2), 100–108. PMC2868556 20467546

[r40] Pillay J, Boylan K, Carrey N, Newton A, Vandermeer B, Nuspl M, MacGregor T, Jafri SHA, Featherstone R and Hartling L (2017) AHRQ comparative effectiveness reviews. In First- and Second-Generation Antipsychotics in Children and Young Adults: Systematic Review Update. Rockville, MD: Agency for Healthcare Research and Quality (US). 28749632

[r41] Raz R, Weisskopf MG, Davidovitch M, Pinto O and Levine H (2015) Differences in autism spectrum disorders incidence by sub-populations in Israel 1992-2009: A total population study. Journal of Autism and Developmental Disorders 45(4), 1062–1069. 10.1007/s10803-014-2262-z. 25287899 PMC4369159

[r42] Righi G, Benevides J, Mazefsky C, Siegel M, Sheinkopf SJ and Morrow EM (2018) Predictors of inpatient psychiatric hospitalization for children and adolescents with autism spectrum disorder. Journal of Autism and Developmental Disorders 48(11), 3647–3657. 10.1007/s10803-017-3154-9. 28536960 PMC5924458

[r43] Russell G, Steer C and Golding J (2011) Social and demographic factors that influence the diagnosis of autistic spectrum disorders. Social Psychiatry and Psychiatric Epidemiology 46(12), 1283–1293. 10.1007/s00127-010-0294-z. 20938640

[r44] Sanders SJ, He X, Willsey AJ, Ercan-Sencicek AG, Samocha KE, Cicek AE, Murtha MT, Bal VH, Bishop SL and Dong S (2015) Insights into autism spectrum disorder genomic architecture and biology from 71 risk loci. Neuron 87(6), 1215–1233. 10.1016/j.neuron.2015.09.016. 26402605 PMC4624267

[r45] Siegel M and Gabriels RL (2014) Psychiatric hospital treatment of children with autism and serious behavioral disturbance. Child and Adolescent Psychiatric Clinics of North America 23(1), 125–142. 10.1016/j.chc.2013.07.004. 24231172

[r46] Smile S (2016) Case 3: Regression in an adolescent with autism spectrum disorder. Paediatrics & Child Health 21(1), 13–14. 10.1093/pch/21.1.13. PMC4758419 26941553

[r47] Srivastava AK and Schwartz CE (2014) Intellectual disability and autism spectrum disorders: Causal genes and molecular mechanisms. Neuroscience and Biobehavioral Reviews 46 Pt 2, 161–174. 10.1016/j.neubiorev.2014.02.015. 24709068 PMC4185273

[r48] Taylor BJ, Sanders KB, Kyle M, Pedersen KA, Veenstra-Vanderweele J and Siegel M (2019) Inpatient psychiatric treatment of serious behavioral problems in children with autism spectrum disorder (ASD): Specialized versus general inpatient units. Journal of Autism and Developmental Disorders 49(3), 1242–1249. 10.1007/s10803-018-3816-2. 30465295

[r49] Thurm A, Farmer C, Salzman E, Lord C and Bishop S (2019) State of the field: Differentiating intellectual disability from autism spectrum disorder. Frontiers in Psychiatry 10, 526. 10.3389/fpsyt.2019.00526. 31417436 PMC6683759

[r50] Tian J, Gao X and Yang L (2022) Repetitive restricted behaviors in autism spectrum disorder: From mechanism to development of therapeutics. Frontiers in Neuroscience 16, 780407. 10.3389/fnins.2022.780407. 35310097 PMC8924045

[r51] Tsakanikos E, Costello H, Holt G, Sturmey P and Bouras N (2007) Behaviour management problems as predictors of psychotropic medication and use of psychiatric services in adults with autism. Journal of Autism and Developmental Disorders 37(6), 1080–1085. 10.1007/s10803-006-0248-1. 17053989

[r52] Tuchman R and Cuccaro M (2011) Epilepsy and autism: Neurodevelopmental perspective. Current Neurology and Neuroscience Reports 11(4), 428–434. 10.1007/s11910-011-0195-x. 21424677

[r53] Zhu X, Need AC, Petrovski S and Goldstein DB (2014) One gene, many neuropsychiatric disorders: Lessons from mendelian diseases. Nature Neuroscience 17(6), 773–781. 10.1038/nn.3713. 24866043

