# Supplementary material for: Characterizing the clinical and sociodemographic profiles of hospitalized adolescents with autism spectrum disorder
Source: Glob Ment Health (Camb). 2024 May 13;11:e63. doi: 10.1017/gmh.2024.63 (PMC11140491; doi:10.1017/gmh.2024.63)
Supplement: Peer review history

# Editor report: Recommendation: Characterizing the clinical and sociodemographic profiles of hospitalized adolescents with autism spectrum disorder — R0/PR2

**DOI:** 10.1017/gmh.2024.63.pr2

**Affiliations:** Psychiatry, Makerere University CHS, Uganda

The authors need to address the comments from review 1 and 2 and resubmit

# Editor report: Decision: Characterizing the clinical and sociodemographic profiles of hospitalized adolescents with autism spectrum disorder — R0/PR3

**DOI:** 10.1017/gmh.2024.63.pr3

**Affiliations:** London School of Hygiene & Tropical Medicine, United Kingdom of Great Britain and Northern Ireland

No accompanying comment.

# Editor report: Recommendation: Characterizing the clinical and sociodemographic profiles of hospitalized adolescents with autism spectrum disorder — R1/PR5

**DOI:** 10.1017/gmh.2024.63.pr5

**Affiliations:** Psychiatry, Makerere University CHS, Uganda

No accompanying comment.

# Editor report: Decision: Characterizing the clinical and sociodemographic profiles of hospitalized adolescents with autism spectrum disorder — R1/PR6

**DOI:** 10.1017/gmh.2024.63.pr6

**Affiliations:** London School of Hygiene & Tropical Medicine, United Kingdom of Great Britain and Northern Ireland

No accompanying comment.
